# Transposon-Mediated Transgenesis in the Short-Lived African Killifish *Nothobranchius furzeri*, a Vertebrate Model for Aging

**DOI:** 10.1534/g3.111.001271

**Published:** 2011-12-01

**Authors:** Dario Riccardo Valenzano, Sabrina Sharp, Anne Brunet

**Affiliations:** Department of Genetics, Stanford University, Stanford, California 94305

**Keywords:** longevity, *Nothobranchius furzeri*, transgenesis, aging model, *Tol2* transposase, killifish

## Abstract

The African killifish *Nothobranchius furzeri* is the shortest-lived vertebrate that can be bred in captivity. *N. furzeri* comprises several wild-derived strains with striking differences in longevity ranging from 3 to 9 months, which makes it a powerful vertebrate model for aging research. The short life cycle of *N. furzeri* should also facilitate studies on adult traits that are specific to vertebrates. Although progress has been made to generate a genetic linkage map and to start sequencing the genome of *N. furzeri*, tools to genetically manipulate this species of fish have not yet been developed. Here, we report the first establishment of transgenesis in *N. furzeri*. We use the *Tol2* transposase system to generate transgenic *N. furzeri* that express green fluorescent protein driven by the *Xenopus* cytoskeletal actin promoter or the zebrafish heat-shock protein 70 promoter. We successfully generate stable transgenic lines of *N. furzeri* with germline transmission of integrated transgene. The development of transgenesis in *N. furzeri* provides a powerful tool to investigate the mechanisms underlying aging and longevity in a short-lived vertebrate model. Transgenesis in this fish will also facilitate the study of other phenotypes, including adult tissue regeneration and cognitive behavior.

The African killifish *Nothobranchius furzeri* (*N. furzeri*) has an exceptionally short lifespan (3–9 months, depending on the strain), and it is a uniquely promising model system for genetic studies of aging and age-dependent diseases in vertebrates (Di Cicco *et al.* 2010; Genade *et al.* 2005; Hartmann *et al.* 2009). These fish normally live in ephemeral water pools in southeastern Africa, where water is present only during the brief rainy season (Genade *et al.* 2005). In the laboratory, the lifespan of *N. furzeri* is 5 to 10 times shorter than the lifespan of mice and zebrafish, respectively. Interestingly, there are several wild-derived *N. furzeri* strains that differ strikingly in their captive lifespan by 2- to 3-fold (Terzibasi *et al.* 2008), potentially allowing the identification of novel genes regulating lifespan in vertebrates by linkage mapping and comparative genomics. *N. furzeri* is also responsive to environmental stimuli that affect aging in other species, including dietary restriction (Terzibasi *et al.* 2009), a resveratrol-rich diet (Valenzano *et al.* 2006b), and temperature (Valenzano *et al.* 2006a). These characteristics make *N. furzeri* an ideal model for research on vertebrate aging and longevity.

Additional features of *N. furzeri* make it an attractive model, even outside the aging field. *N. furzeri* can achieve sexual maturation in 25–30 days, providing a unique system to study the developmental processes involved in maturation to adulthood. Under controlled laboratory conditions, *N. furzeri* has the shortest life cycle among vertebrate species that can be bred in captivity (about 40 days), which should facilitate the study of adult traits, including tissue regeneration, cognitive behavior, and susceptibility to disease. Finally, like other species of the genus *Nothobranchius*, *N. furzeri* undergoes embryonic diapause, with embryos that can survive in dry mud for up to a year (Levels *et al.* 1986; Markofsky and Matias 1977; Matias 1984), providing a potential model for developmental diapause in vertebrates.

Several genetic and genomic tools have already been developed in *N. furzeri*. The first genetic linkage map based on microsatellites was recently generated in this species of fish (Valenzano *et al.* 2009). Furthermore, *N. furzeri*’s genome has been partially sequenced by shotgun Sanger sequencing (Reichwald *et al.* 2009). Next-generation sequencing of the genome of short-lived and long-lived strains of *N. furzeri* is underway. However, no method of transgene expression has been developed to date in this species. Transgenesis in a vertebrate model with a short lifespan and life cycle would be a powerful tool to screen for genes that govern adult phenotypes.

Transgenesis in fish has greatly benefited from transposase systems, such as *Tol1*, *Tol2*, and *Sleeping Beauty*, which were all successfully used in zebrafish transgenesis (Davidson *et al.* 2003; Grabher and Wittbrodt 2007; Kawakami *et al.* 2000, 2004; Koga *et al.* 2008). The *Tol2* transposon was also developed as a system to efficiently generate transgenic animals in other model systems, including stickleback, the frog *Xenopus tropicalis*, and chicken (Chan *et al.* 2010; Hamlet *et al.* 2006; Kawakami 2007). *Tol2* is an active DNA transposable element capable of catalyzing transposition upon recognition of a target sequence. The *Tol2* transposition system used for transgenesis consists of two elements: an RNA encoding the *Tol2* transposase and a plasmid containing a nonautonomous *Tol2* transposon (*i.e.* not encoding the transposase) surrounding the gene of interest (Kawakami 2004, 2007; Kawakami *et al.* 2000). The transposase recognizes the target *Tol2* sequence in the plasmid, excises the gene of interest, and integrates it into the host’s genome (Kawakami *et al.* 2000; Ni *et al.* 2008), thus allowing efficient and stable transgenesis.

Here, we generate transgenic *N. furzeri* fish expressing green fluorescent protein (GFP) under the control of the ubiquitous *Xenopus* cytoskeletal actin promoter or the zebrafish heat-shock protein 70 promoter using the *Tol2* transposase system. We also show that the transgene is stably integrated into the genome and can be transmitted through the germline to the F1 and F2 generations. The development of transgenesis in *N. furzeri* will be crucial for testing the role of specific genes in the shortest-lived available vertebrate model system.

## Material and Methods

### Constructs

**Figure 1:**
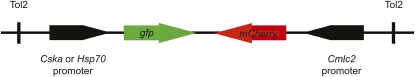
Transgenic constructs. Transgenic constructs containing two *Tol2* recognition elements (1 and 2) flanking a cassette comprising a promoter driving the *gfp* reporter gene. Two promoters were used in this study: *Xenopus borealis cytoskeletal actin* (*Cska*) and zebrafish *heat shock protein 70* (*Hsp70*). This cassette also contains the zebrafish *cardiac myocyte light chain* (*Cmlc2*) promoter driving the *mCherry* gene, although we have not analyzed expression of *mCherry*. The *Tol2* transposase recognizes the *Tol2* elements, excises the cassette, and integrates it into the host’s genome.

The pBHR construct expressing GFP under the control of the zebrafish *heat-shock protein 70* (*Hsp70*) promoter was obtained from David Kingsley (Chan *et al.* 2010) and was renamed *pHsp70-gfp Tol2* in the remainder of the study. The *pCska-gfp Tol2* construct was generated by subcloning the *Xenopus borealis cytoskeletal actin* promoter from the *pCska-gfp-SceI* construct (Thermes *et al.* 2002) into the *pHsp70-gfp Tol2* (pBHR) construct (Chan *et al.* 2010) between the *Apa*I and *Sal*I sites, thereby replacing the *Hsp70* promoter cassette (Figure 1). The *pHsp70-gfp Tol2* and *pCska-gfp Tol2* constructs also contain the *mCherry* reporter gene driven by the zebrafish *cardiac myocyte light chain* (*Cmlc2*) promoter.

### RNA synthesis of *Tol2* transposase mRNA

The medaka *Tol2* transposase mRNA was transcribed from the pCS-TP plasmid (Kawakami *et al.* 2004) using the mMESSAGE mMACHINE SP6 kit (Ambion, Austin, TX), according to the manufacturer’s protocol.

### Fish husbandry

*N. furzeri* strains [GRZ and MZM-0403 (Terzibasi *et al.* 2008)] were housed at 25° in a central filtration recirculating system with a 12 hr light/dark cycle as previously described (Genade *et al.* 2005; Valenzano *et al.* 2009) and fed twice a day with freeze-dried bloodworms (Hikari, Japan).

### Production of injectable *N. furzeri* embryos

*N. furzeri* embryos can undergo diapause, a developmental arrested stage that can last up to a year (Inglima *et al.* 1981; Podrabsky and Hand 1999). Prolonged exposure of the fertilized embryos to low oxygen or to the presence of adult fish induces diapause (Inglima *et al.* 1981), thereby substantially lengthening the life cycle. To produce one- to two-cell stage embryos for microinjection and to avoid diapause initiation, one 4- to 5-week-old fertile adult male (*i.e.* one that has already generated fertilized eggs) was placed with three fertile females of the same age in the same 9.5 liter tank. Male and female fish were separated by a plastic mesh for 24 hr. The night prior to injection, the mesh was removed and the fish were allowed to naturally spawn overnight over a fine sand substrate until 2 hr after the light was turned on in the fish room. Eggs were sieved from the sand with a strainer and kept at 4° in 1X Yamamoto embryo solution (17 mM NaCl, 2.7 mM KCl, 2.5 mM CaCl_2_, 0.02 mM NaHCO_3_, pH 7.3) (Westerfield 2000) with 0.1 μl/ml of Methylene Blue (Kordon, 2.3% stock solution) to limit parasitic infection. Injections occurred within 1 hr of embryo collection. With this protocol, 18% of the embryos are at the one-cell stage, 28% are at the two-cell stage, and 54% have more than two cells (n = 303 embryos). Embryos were injected at the one- or two-cell stage to minimize chimeric integration of the transgene.

### Injection of *N. furzeri* embryos

**Figure 2:**
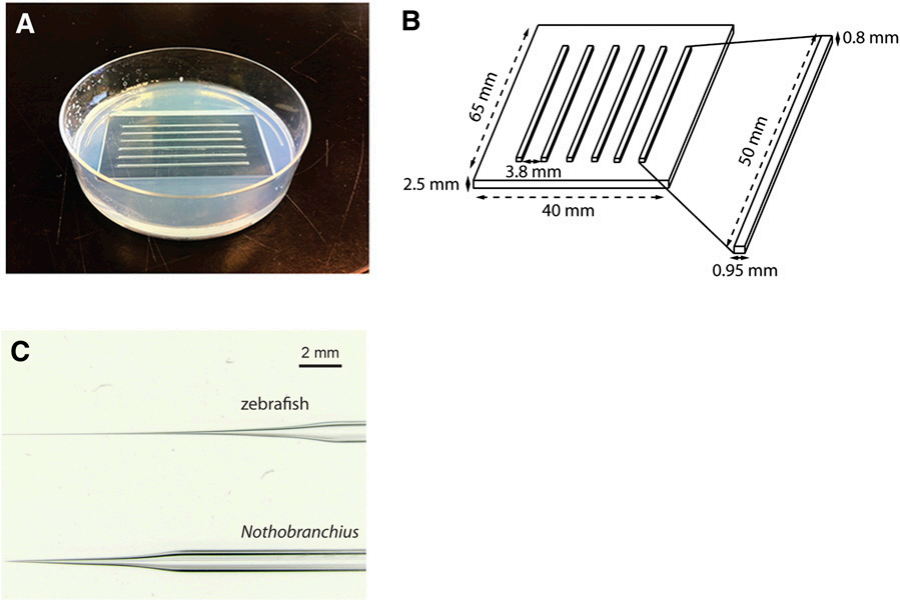
Microinjection plate and needle for transgenesis. (A) Microinjection plate cast using an *ad hoc* built plastic mold. Embryos selected for injection are aligned along the trenches and pierced with borosilicate needles. Up to 40 embryos could fit each trench. (B) Plastic mold design and measures. (C) Comparison between the microinjection needle for zebrafish (top) and *N. furzeri* (bottom). *N. furzeri* needles are sturdier than zebrafish needles, allowing better chorion piercing.

To hold the embryos for injection, a 1.5% agarose plate was cast with an *ad hoc* built plastic mold that produced six 1 mm-wide trenches (Figure 2, A and B). One- or two-cell stage embryos were injected using borosilicate microcapillaries (Harvard Apparatus GC100F-10, Holliston, MA). Capillaries were pulled with a micropipette puller (Sutter Instrument P-87, Novato, CA) using the following parameters: pressure = 450, heat = ramp value − 15°; pull = 50; velocity = 80; time = 200. The needles designed for *N. furzeri* injection have a shorter and less flexible tip than zebrafish injection needles to allow easier penetration through a thicker chorion (Figure 2C). The capillary was then filled with 1.5 μl of a water solution containing 15 ng/μl of plasmid DNA (purified by Plasmid Maxi kit, Qiagen), 15 ng/μl of *Tol2* transposase RNA, and 1% phenol red (Sigma-Aldrich, Saint Louis, MO) to track the injected solution. The embryos were injected with 5 pl of this solution, which corresponds to a tenth of the embryo’s volume at the one- or two-cell stage. The solution was pressure-injected at 30 psi with 75 ms pulses. Injections were performed under a Nikon C-PS stereoscope and Zeiss KL 1500 LCD (Stuttgart, Germany) optic fibers. The injection apparatus (Applied Scientific Instrumentation, Eugene, OR) consists of an MHC model magnetic stand, an MMPI model pressure injector, a foot switch to pulse the injected solution into the embryos, an MM3 model micromanipulator, and an M-PIP model micropipette holder (Applied Scientific Instrumentation), assisted by a backpressure unit (Warner Instrument, Hamden, CT) to gauge the pulsed release of pressurized nitrogen.

### Development of injected embryos

Injected and control noninjected embryos were immersed in 1× Yamamoto embryo solution with 0.1 μl/ml of Methylene Blue and incubated at 26° until they developed eyes (6–8 days). Upon development of the eyes, embryos were inspected for GFP fluorescence (see below) and transferred to Petri dishes with autoclaved, humidified, nonchemically treated peat moss substrate (Uni-Gro Premium Organic Peat Moss, San Bernardino, CA). Petri dishes were sealed with parafilm and incubated at room temperature until completion of fish development (additional 5–10 days).

### Detection of GFP expression by fluorescence microscopy

To detect GFP fluorescence in injected fish and their offspring, a Leica (Wetzlar, Germany) MZ-16 FA fluorescence stereomicroscope was used. Images were acquired using the following parameters for GFP: 1.1 sec of exposure; 2.1× of gain; 0.9 of saturation; 0.53 of gamma; and for bright field: 97 ms of exposure; 2.1× of gain; 0.9 of saturation; 0.53 of gamma. We used 2.1 sec of exposure to detect GFP in *pHsp70-gfp Tol2* embryos. Images were acquired with a Leica DFC 500 camera and processed with Leica application Suite V3.

### PCR on genomic DNA

Germline transmission of the transgene was tested by PCR on genomic DNA extracted from caudal fin clips following a standard zebrafish genomic DNA extraction protocol (Westerfield 2000). PCR reactions were performed in 25 μl of reaction volume with 1.25 units of Taq DNA polymerase (ABI AmpliTaq, Foster City, CA), 1× PCR buffer (ABI AmpliTaq), 2.5 mM MgCl_2_, 5 ng of genomic DNA, 0.8 μM dNTPs (Invitrogen, Carlsbad, CA), 0.4 μM of primers (*gfp* forward primer, 5′ CAC ATG AAG CAG CAC GAC TT 3′; *gfp* reverse primer, 5′ TGC TCA GGT AGT GGT TGT CG 3′; *N. furzeri igf1r* forward primer, 5′ CCA TCC TAG CGA CTA TCT TAA TTG T 3′; *N. furzeri igf1r* reverse primer, 5′ TCC TTA ACA ACG CCT TTC G 3′).

The following program was used: 95° for 3 min, 5 cycles of 30 sec at 95°, 30 sec at 57°, 45 sec at 72°, 27 cycles of 30 sec at 95°, 30 sec at 56°, 45 sec at 72°, and 4 min at 72°. PCR products (379 bp for *gfp* and 550 bp for *igf1r*) were resolved on a 1.5% agarose gel.

### Southern blot analysis

For Southern blot analysis, 5 μg of caudal fin was digested with *Bam*HI and *Eco*RI for 16 hr. Digested DNA was resolved on a 0.7% agarose gel, depurinated in 0.2 M HCl, denatured in Denaturing Buffer (0.5 M NaOH, 1.5 M NaCl), and transferred to a Hybond-N nylon membrane (GE Healthcare Life Sciences) by capillary transfer in SSC 20× buffer (Invitrogen). The membrane was hybridized overnight at 66° with a ^32^P-labeled probe in QuickHyb solution (Stratagene, La Jolla, CA). The membrane was washed twice for 20 min in 2× SSC buffer, 0.1% SDS, and then twice in 0.2× SSC buffer, 0.1% SDS before exposure (Amersham Hyperfilm MP, GE Healthcare Life Sciences).

The probe was generated by PCR amplification of a 379 bp region within the *gf*p cDNA from the *pCska-gfp Tol2* plasmid (1 ng). The PCR conditions were the same as those described above. The PCR product was resolved on a 1% agarose gel, and the *gfp* DNA fragment was purified using a Qiaquick gel extraction kit (Qiagen). The probe was labeled using the RadPrime DNA Labeling kit (Invitrogen) and 30 μCi of α^32^P dGTP (PerkinElmer, Waltham, MA). The labeled probe was purified on a G50 microspin column (GE Healthcare Life Sciences), denatured for 5 min at 95°, and added to the membrane for hybridization.

## Results

The African killifish *N. furzeri* is the shortest-lived, fastest maturing vertebrate that can be reproduced in captivity (Table 1). It is a uniquely promising new vertebrate model system to study aging and age-dependent traits. A critical step in developing *N. furzeri* as a model system is the ability to generate transgenic fish.

**Table 1:** *N. furzeri* displays the shortest maturation time, lifespan, and life cycle among fish models

Species	Time to Hatching	Sexual Maturation	Lifespan	Life Cycle
Zebrafish*^a^*	2–3 days	60–75 days	3–5 years	60–75 days
Medaka*^b^*	∼9 days	60–75 days	3–5 years	70–80 days
Stickleback*^c^*	∼8 days	60 days to 6 months	3–5 years	68 days to 6 months
*N. furzeri*	12–18 days*^d^*	25–30 days	0.25–0.75 years	≥37 days

Cross-species comparison of life history traits under controlled laboratory conditions.

a Westerfield (2000).

b Egami and Etoh (1969); Iwamatsu (2004); Takeda and Shimada (2010).

c Swarup (1958); Wootton (1984).

d In embryos that do not enter diapause.

### Development of a microinjection system adapted to the thick chorion of *N. furzeri* embryos

*N. furzeri* embryos are protected by a thick chorion that allows them to survive prolonged drought and develop in the absence of water. To pierce through the thick chorion, we designed specific injection needles that are shorter and less flexible than the standard needles used to inject zebrafish eggs, and we clamped the eggs within agarose trenches (Figure 2). To increase the chance of germline transmission of the transgene, we injected *N. furzeri* embryos at the one- or two-cell stage.

### The *Tol2* transposase system allows efficient GFP expression in *N. furzeri* embryos from two different promoters

On the basis of successful transgenesis methods developed in zebrafish and stickleback (Chan *et al.* 2010; Kawakami *et al.* 2000), we used the *Tol2* transposase system. We generated a *pCska-gfp Tol2* construct encoding enhanced GFP driven by the *Xenopus* cytoskeletal actin promoter, a promoter that has been shown to be active in *Xenopus* and zebrafish and allows ubiquitous expression (Figure 1) (Lakin *et al.* 1993; Thermes *et al.* 2002). The *pCska-gfp Tol2* construct was injected into one- or two-cell stage embryos of the MZM-0403 strain of *N. furzeri* in the presence or absence of *Tol2* transposase mRNA. The expression of GFP in live embryos was determined by fluorescence stereomicroscopy at 12 days postinjection. The presence of the *Tol2* mRNA increases the percentage of GFP-positive fish from 0.8% (1 out of 127) to 25% (15 out of 61). We also injected the *pHsp70-gfp Tol2* construct (Chan *et al.* 2010) to drive GFP expression from the zebrafish *Hsp70* promoter (Figure 1). With the *pHsp70-gfp Tol2* construct, 36% of the embryos were positive for GFP (33 out of 92) in the presence of *Tol2* mRNA. These results indicate that the presence of the *Tol2* transposase allows expression of the transgene in about one third of the embryos that survived injection (at this stage, our percentage of survival after injection is 70%).

### Expression of GFP from the *cytoskeletal actin* and the *hsp70* promoters is ubiquitous and persists until adulthood

**Figure 3:**
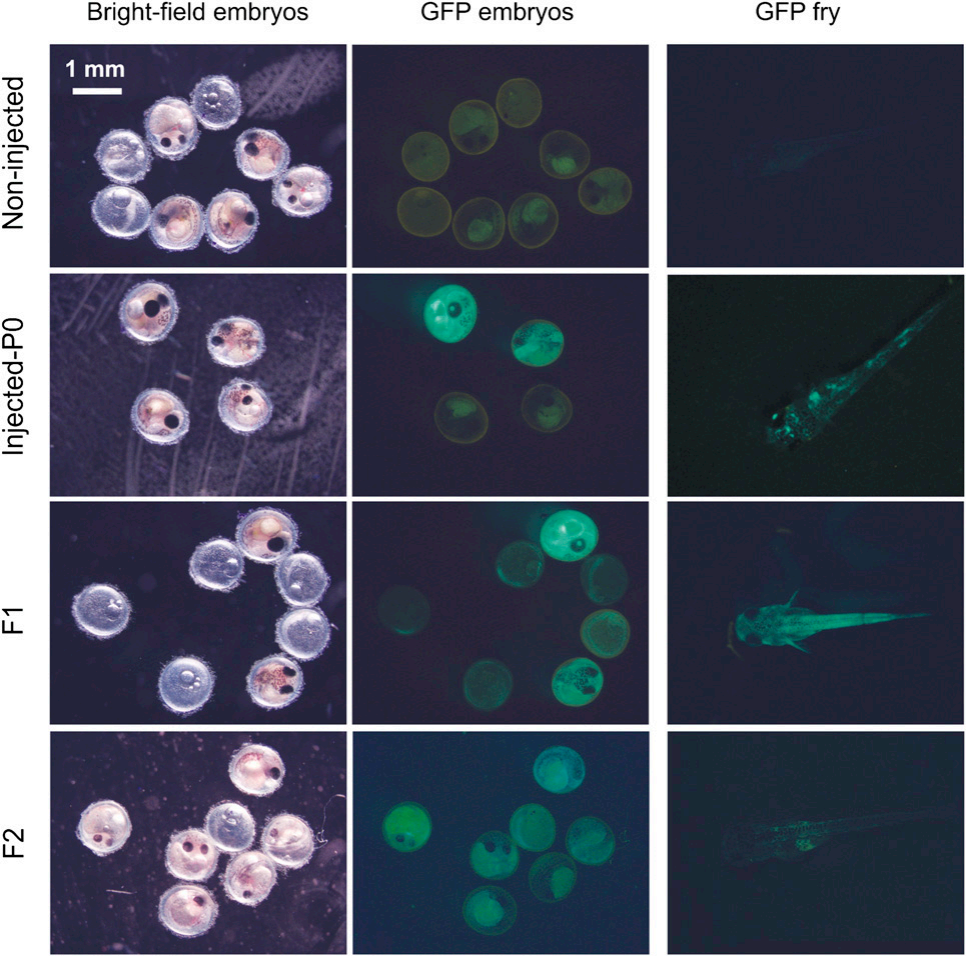
Expression of GFP in *pCska-gfp Tol2* transgenic *N. furzeri*. GFP expression in live noninjected (top row), injected P0 fish (second row), and the F1 (third row), and F2 (bottom row) progeny of GFP-positive *N. furzeri*. Pictures were taken 12 days postfertilization for embryos, and 5 days posthatching for fry. Bright field and GFP images are shown for embryos. Scale bar: 1 mm. GFP, green fluorescent protein.

**Figure 4:**
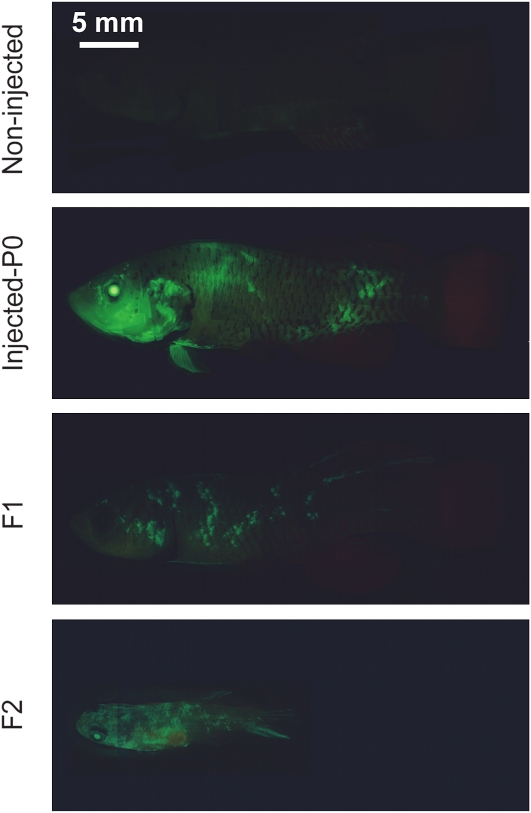
Expression of GFP in adult *N. furzeri* injected with *pCska-gfp Tol2*. GFP expression in noninjected P0 adult fish and in P0, F1, and F2 adult fish injected with the *pCska-gfp Tol2* construct. The P0 and F1 fish are 3-month-old adults. The F2 fish is a 1-month-old adult. The GFP images for individual fish were digitally assembled from individual snapshots. Scale bar: 5 mm. GFP, green fluorescent protein.

To determine the pattern of GFP expression in fish injected with the *pCskA-gfp Tol2* construct and to test how long GFP expression lasted, we monitored GFP expression in live, injected 12-day-old embryos, 5-day-old fry, and 1- to 3-month-old adult fish by fluorescence stereomicroscopy. Embryos and fry that were injected with the *pCskA-gfp Tol2* construct showed strong GFP expression (Figure 3). The expression of GFP was patchy in fry, probably due to a certain degree of mosaicism (Figure 3). Adult fish expressed GFP relatively ubiquitously, with high expression in the eyes (Figure 4). These results indicate that expression of the transgene persists through adulthood, suggesting that the transgene has integrated into *N. furzeri*’s genome.

**Figure 5:**
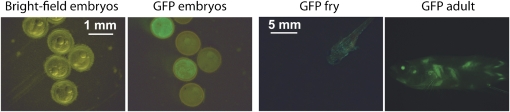
Expression of GFP in *N. furzeri* injected with *pHsp70-gfp Tol2*. GFP expression in 12-day-old embryos, 5-day-old fry, and 3-month-old adult *N. furzeri* fish injected with the *pHsp70-gfp Tol2* construct. GFP expression was observed even without heat-shocking the individuals. Bright field and GPF images are shown for embryos. Scale bar: 1 mm for embryos; 5 mm for fry and adult fish. GFP, green fluorescent protein.

We also monitored GFP expression in fish injected with the *pHsp70-gfp Tol2* transgenic construct. Fish injected with this construct expressed GFP in embryo, fry, and adult (Figure 5). GFP expression was detected even in the absence of heat shock, likely due to the basal activity of the *hsp70* promoter. Thus, the *Tol2* transposase system allowed us to generate P0 *N. furzeri* fish that express GFP from two different promoters.

### Generation of transgenic lines expressing GFP from the *cytoskeletal actin* promoter in *N. furzeri*

One of the most important aspects of transgenesis is the ability to generate lines of transgenic fish by achieving germline transmission of the transgene. To determine whether the parental transgenic fish could transmit the transgene to their progeny, we monitored GFP expression in F1 offspring of five GFP-positive male P0 fish injected with *pCska-gfp Tol2* and positive for GFP expression, each crossed with 3 wild-type adult females. Of the 5 tested males, 2 produced transgenic offspring, and 130 out of 452 F1 fish (29%) were GFP-positive by fluorescence stereomicroscopy (Figures 3 and 4). These results indicate that the transgene can be successfully transmitted through the germline. The fact that less than 50% of the F1 offspring of P0 GFP-positive fish are GFP-positive could be due to mosaicism of the integration or to silencing of the transgene. Intercrossing GFP-positive F1 fish resulted in GFP-positive F2 transgenic offspring (Figures 3 and 4). Thus, the *Tol2* transposase system allows germline transmission of the transgene.

### Genomic integration of the transgene

**Figure 6:**
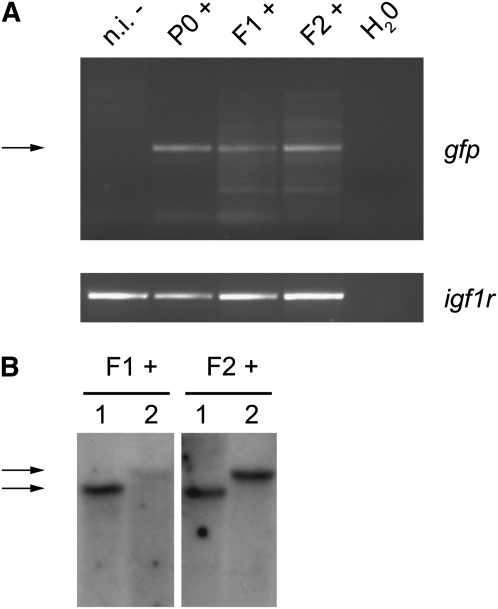
Integration of the transgene into *N. furzeri*’s genome (A) PCR amplification of genomic DNA from transgenic fish using primers for *gfp* (upper panel) and *igf1r* (lower panel). The arrow indicates the amplified *gfp* band. F1+: F1 GFP-positive fish generated by crossing the P0 GFP-positive individual with a wild-type fish; F2+: F2 GFP-positive fish generated by intercrossing two F1 GFP-positive fish. n.i., noninjected control fish. (B) Southern blot on genomic DNA from transgenic fish using a probe for *gfp*. F1+: two F1 GFP-positive fish generated by crossing the P0 GFP-positive individual with a wild-type fish; F2+: two F2 GFP-positive fish generated by intercrossing two F1 GFP-positive fish. F1 and F2 fish with the same number (1 and 2) belong to the same family and, therefore, share the same band size (indicated by the arrows) for *gfp*. GFP, green fluorescent protein.

To test whether the transgene is chromosomally transmitted through the germline, we analyzed genomic DNA from GFP-positive P0 fish, GFP-positive F1 and F2 fish, and noninjected fish using PCR with primers specific to *gfp*. We found that genomic DNA of GFP-positive P0, F1, and F2 fish indeed contained the *gfp* DNA (Figure 6A). These results suggest that the transgene is integrated into the genome of *N. furzeri* and chromosomally transmitted through the germline.

To test in an independent manner the integration of the transgene into *N. furzeri*’s genome, we analyzed the genomic DNA of two F1 and F2 GFP-positive individuals from the same P0 parent by Southern blot with a *gfp* probe. We found that F1 and F2 fish genomic DNA contains bands that are recognized by the *gfp* probe (Figure 6B). There were two distinct banding patterns, suggesting at least two integration sites of the transgene (Figure 6B). Analysis of two other F2 fish from the same parent by Southern blot revealed two additional banding patterns (data not shown). Collectively, these results indicate that the transgene is integrated in the genome of *N. furzeri* and that it can be chromosomally transmitted through the germline. The presence of several banding patterns in progeny from one P0 parent indicates that *Tol2* transgenesis in *N. furzeri* resulted in several integration sites in the P0 injected fish. The successful development of transgenesis in an exceptionally short-lived vertebrate will be a crucial step in rapidly screening for genes that regulate lifespan and adult traits in vertebrates.

## Discussion

In this study, we successfully developed transgenesis in the African short-lived killifish *N. furzeri* using the *Tol2* transposase system. We generated transgenic lines of *N. furzeri* expressing GFP under the control of a *Xenopus* ubiquitous cytoskeletal actin promoter, with integration of the transgene into the genome and successful chromosomal transmission of the transgene trough the germline. Along with the *I-SceI* meganuclease system and the *Sleeping Beauty* transposase system (Grabher and Wittbrodt 2007), *Tol2* is the most effective transgenesis tool used in fish (Kawakami 2007; Ni *et al.* 2008). The *Tol2* transposase system has been widely used in zebrafish to create stable lines of transgenic fish (Kawakami 2004; Suster *et al.* 2009). This system has also been used with success in stickleback to generate transgenic P0 individuals (Chan *et al.* 2010). Transgenic lines have only been developed in laboratory fish species, such as zebrafish and medaka (Lu *et al.* 1992; Stuart *et al.* 1988), or in commercially relevant species such as salmon, trout, and tilapia (Guyomard *et al.* 1989; Martinez *et al.* 1996; Yaskowiak *et al.* 2006). Our study adds *N. furzeri* to the fish species that can be used as genetically tractable models to study specific traits.

The percentage of injected *N. furzeri* embryos that are GFP-positive as visualized by fluorescence microscopy is relatively high (one quarter to one third of the injected *N. furzeri* embryos, depending on the construct injected). The percentage of embryos that have integrated the transgene may even be higher. Construct integration in transcriptionally inactive regions could prevent transgene expression. Active silencing of the transcript or targeted GFP protein degradation could also lower the overall number of transgenic fish that visibly express GFP. Differences in GFP expression rates between the two constructs (*pCska-gfp Tol2* and *pHsp70-gfp Tol2*) may be due to the promoters used, which likely promote different pattern and intensity of expression, thereby affecting the threshold for detecting GFP fluorescence.

GFP expression driven by the *cytoskeletal actin* (*Cska*) promoter or the *heat-shock protein 70* (*Hsp70*) promoter is relatively ubiquitous in *N. furzeri* in the P0 generation. Expression of GFP from the *hsp70* promoter was observed even in the absence of heat shock, possibly because this promoter allows basal level of expression. Alternatively, fish may be experiencing some levels of stress that trigger a heat-shock–like response. As both transgenes also contain the mCherry reporter gene under the control of the *cardiac myocyte light chain* (*Cmlc2*) promoter, we anticipate that transgenic fish should also express mCherry in their heart, although we have not examined the pattern of expression of mCherry. The pervasive expression of GFP in P0 individuals could be due to the early integration of the transgene during development and/or to multiple integration sites of the transgene in the genome. Embryonic development is slower in *N. furzeri* than in zebrafish, which may facilitate early integration of the transgene and germline transmission. The slow embryonic development of *N. furzeri* might increase the chances of early embryo integration and, therefore, robust germline transmission compared with other model systems. Indeed, the frequency of GFP-positive F1 offspring from a cross between GFP-positive P0 parents and wild-type fish is about 30% in *N. furzeri*. A perfect germline transmission with one site of integration would give rise to 50% GFP-positive F1 fish. Given that there is more than one integration site in P0 parents (Figure 6B), the fact that we obtained 30% GFP-positive F1 fish indicates that there is a certain degree of mosaicism of the germline but that germline transmission is relatively efficient. As a comparison, the frequency of transgenic F1 offspring has been reported to range from 3 to 100% in zebrafish (Kawakami *et al.* 2004; Urasaki *et al.* 2006). Thus, *N. furzeri* germline transmission of the transgene is a relatively frequent event and is well within the range of that of zebrafish, which should highly facilitate the generation of transgenic lines.

The development of transgenesis in *N. furzeri*, together with the recent development of genetic and genomic resources for this species (Reichwald *et al.* 2009; Valenzano *et al.* 2009), provides the scientific community with a powerful new model system to study vertebrate aging. For example, transgenesis in *N. furzeri* will help to rapidly test the effects of candidate genes in modulating vertebrate longevity, and it opens the possibility of genetic screens for genes affecting aging in vertebrates. The use of transgenesis in *N. furzeri* will also help study several other vertebrate-specific phenotypes. For example, it could facilitate identification of the mechanisms underlying tissue regeneration and adult stem-cell function in relation to age. Moreover, *N. furzeri* transgenesis will be helpful to analyze the ontogenesis of conspicuous sexual traits, such as fin pigmentation patterns and courtship behaviors in males. Finally, transgenesis will be a key tool in identifying genetic factors regulating developmental diapause, a characteristic phenotype of oviparous fish of the order Cyprinodontiformes, which includes the genus *Nothobranchius* (Inglima *et al.* 1981; Levels *et al.* 1986; Markofsky and Matias 1977; Matias 1984; Podrabsky *et al.* 2001; Podrabsky and Somero 2007). Transgenesis is a crucial step in the development of *N. furzeri* as a genetically tractable short-lived vertebrate system.
